# Characterization and evaluation of anti-*Salmonella enteritidis* activity of indigenous probiotic lactobacilli in mice

**DOI:** 10.1515/biol-2022-0100

**Published:** 2022-08-17

**Authors:** Amina Mustafa, Muhammad Nawaz, Masood Rabbani, Muhammad Tayyab, Madiha Khan

**Affiliations:** Institute of Microbiology, University of Veterinary and Animal Sciences, Lahore, 54000, Punjab, Pakistan; Research School of Biology, Australian National University, Canberra, 2601, ACT, Australia; Institute of Biochemistry and Biotechnology, University of Veterinary and Animal Sciences, Lahore, 54000, Punjab, Pakistan; Department of Microbiology, University of Central Punjab, Lahore, 54000, Punjab, Pakistan

**Keywords:** lactobacilli, *Salmonella enteritidis*, mice, yogurt, human infants

## Abstract

Lactobacilli (*n* = 24), isolated from human infants and yogurt, showed variable *in vitro* activity against *Salmonella enteritidis* (8.0 ± 1.0 to 16.6 ± 0.5 mm) and other gut pathogens (9.0 ± 1.0 to 15.3 ± 0.5 mm), as determined by a well diffusion assay. The isolates were identified as *Limosilactobacillus fermentum* (FY1, FY3, FY4, IL2, and IL5), *Lactobacillus delbrueckii* (FY6 and FY7), *Lactobacillus* sp. (IL7), and *Lactobacillus gasseri* (IL12). All isolates showed variable *in vitro* tolerance to acidic pH for 3 h and visible growth at pH 4 and in the presence of 0.3% ox-bile. The antibiotic susceptibility profile of *Lactobacillus* isolates indicated resistance against vancomycin, ciprofloxacin, streptomycin, and lincomycin. Isolates had variable auto-aggregation and showed variable capabilities to co-aggregate with *S. enteritidis*. Based on all tested parameters, *L. fermentum* IL2, *L. fermentum* IL5, and *L. gasseri* IL12 were selected for co-culture experiments, followed by *in vivo* evaluation in Balb/c mice. All the selected isolates resulted in a 100% reduction in *S. enteritidis* in broth. *Lactobacillus* isolates efficiently colonized mouse guts and inhibited *S. enteritidis* colonization. Overall, there was ≥99.06% and ≤4.32 Mean log_10_ reduction in *Salmonella* counts in mice feces within 7 days. The study, thus, provided characterized lactobacilli that could be considered as potential ingredients for probiotic formulations intended to prevent *S. enteritidis* infection in humans.

## 1 Introduction

*Salmonella enterica* is a leading bacterial cause of foodborne illnesses, causing a considerable burden globally [1]. Out of 2,600 salmonellae, *Salmonella enterica* serovar Enteritidis is a significant pathogen of public health importance [2]. *Salmonella enteritidis* causes gastrointestinal inflammation, with diarrhea as the most common symptom. Diarrhea may cause excessive water loss while removing bacteria from the body. Thus, the innovation of effective control strategies against *Salmonella enteritidis* infections has been a moving target [3]. In Pakistan, it is an endemic pathogen causing regular outbreaks associated with poultry products [4]. Moreover, it can be transmitted both vertically and horizontally in poultry and eventually to humans [5]. In the 20th century, *Salmonella enteritidis* emerged as a major egg-associated pathogen [6]. Although numerous antibiotics are available for treating *Salmonella* infections, their excessive use has mounted anti-microbial resistance, treatment failures, and a negative impact on human health [7]. Isolation of multi-drug resistant *Salmonella enteritidis* from different regions of Pakistan has disclosed a potential threat not only to humans but also to the poultry industry of Pakistan [8]. Therefore, Pakistan has restricted the preventive and medical use of antibiotics in poultry and livestock sectors [9]. However, managing drug-resistant *Salmonella enteritidis* infections by non-antibiotic prophylactic regimens (including probiotics) is an acceptable alternative [10].

Probiotics or direct-fed microbes are currently receiving considerable interest as an alternative to antibiotics. According to World Health Organization and Food and Agriculture Organization, probiotics are the living microorganisms which when administered in adequate amount confer a health benefit to the host [11]. These have been classified into two broad categories, including classical probiotics (*Bifidobacterium*, *Lactobacillus*, and *Saccharomyces boulardii*) and next-generation probiotics (*Faecalibacterium prausnitzii* and *Akkermancia muciniphila*) [12]. The different strains of probiotics are being used in numerous supplements, drugs, food products, milk powder, cheese, yogurt, ice cream, and fruit juices [13].

Probiotics are distinguished for providing health benefits, including immune boosting, protection against gut pathogens, binding of mycotoxins, strengthening of gut function and microbiota, and increased absorption of nutrients [14]. It has been hypothesized that probiotic lactobacilli have antibacterial potential and, thus, can prevent the growth of pathogenic bacteria like *Salmonella*, *Escherichia coli,* and *Campylobacter* [15,16]. The anti-*Salmonella* activity of lactic acid bacteria and their potential to act as a probiotic feed additive, especially against salmonellosis, have been demonstrated previously [17]. Currently, the mechanistic action of probiotics is still under consideration. The mechanisms of action of probiotics have not been fully revealed. However, the most favored mechanisms include the production of antibacterial compounds like bacteriocins, organic acids, and H_2_O_2_, a decline in gut pH, competition for nutrients, immune modulation, modulation of cytokine pattern and toxin action, reduction in bacterial enzyme activity, neutralization of enterotoxins, mycotoxin removal by physical binding, competitive exclusion of pathogen, and antagonism of the pathogen, followed by strengthening of normal flora and enhanced digestive activity [18,19].

The anti-microbial effect of probiotics is species and strain-specific, which is more prominent against pathogens from the same origin. Therefore, extensive screening is required to select probiotic strains [20]. Due to the beneficial attributes and lesser side effects, there has been increased interest in searching out novel probiotic strains. Thus, the present work is aimed to isolate anti-*S. enteritidis* lactobacilli, determine their *in vitro* probiotic properties, and evaluate the resultant probiotics for the prevention of *Salmonella enteritidis* colonization in mice.

## 2 Methods

### 2.1 Isolation and identification of lactobacilli

Yogurt samples (*n* = 15), including both homemade (*n* = 7) and commercial (*n* = 8), were collected from different areas of Lahore. Fecal swab samples (*n* = 15) were obtained from healthy humans (breastfed infants) after taking informed consent from their parents/guardians. All samples were transported to the Institute of Microbiology, the University of Veterinary and Animal Sciences (UVAS), Lahore, where these were serially diluted (ten-fold) and plated on de Man, Rogosa, and Sharpe (MRS) agar (Merck, Germany), as described in a previous study [21]. After 24–48 h incubation at 37°C (equivalent to human body temperature) in an anaerobic jar (Oxoid Anaerobic indicator BR0055), individual colonies were purified and stored in MRS broth supplemented with 20% glycerol at −20°C. Lactobacilli were initially identified by Gram’s staining and a catalase test, as described by Bergey’s Manual of Determinative Bacteriology [22]. DNAs of lactobacilli were isolated using a DNA extraction kit (Geneall, South Korea), according to the manufacturer’s instructions. *Lactobacillus* genus was confirmed using specific primers XB5-F (5′-GCCTTGTACACACCGCCCGT-3′) and LbLMA1-R (5′-CTCAAAACTAAACAAAGTTTC-3′) targeting 16S–23S inter-spacer regions, as used earlier [23]. Whereas, 16S rDNAs were amplified using universal primers 8FLP-F (5′-AGTTTGATCNCTGGCTCAG-3′) and XB4-R (5′-GTGTGTACAAGGCCCGGGAAC-3′) [24]. The fragments were amplified in a Bio-Rad T100^TM^ Thermo-cycler using the following program: initial denaturation at 94°C for 10 min, followed by 35 cycles of final denaturation at 94°C for 1 min, annealing at 55°C for 1 min, and initial extension at 72°C for 1 min. In the end, the final extension was performed at 72°C for 10 min. The sequences of 16S rDNA amplicons (∼1400 bp) or 16S–23S rDNA inter-spacer (∼250 bp) were submitted to GenBank, and accession numbers were obtained.


**Informed consent:** Informed consent has been obtained from the guardians of all individuals included in this study.
**Ethical approval:** The research related to human use has been complied with all the relevant national regulations, institutional policies and in accordance with the tenets of the Helsinki Declaration, and has been approved by the authors’ institutional review board or equivalent committee.

### 2.2 Screening of anti-*Salmonella enteritidis* lactobacilli

Lactobacilli were screened for their activity against *Salmonella enteritidis* (D) (ATCC13076^TM^) and antibiotic-resistant salmonellae (*Salmonella enteritidis* 54, *Salmonella enteritidis* 56, *Salmonella enteritidis* 58, and *Salmonella enteritidis* 91) of poultry origin, that were isolated previously by Yasmin [9]. The antibiotic susceptibility patterns of salmonellae are mentioned in Table 1.

**Table 1:** Antibiotic susceptibility patterns of salmonellae

Strain	Antibiotic resistance profile
S.E 54	AMP^R^, AMX^R^, CHL^R^, CIP^R^, NAL^R^, TET^R^, CRO^R^, and CFM^R^
S.E 56	AMP^R^, AMX^R^, CHL^R^, CIP^R^, NAL^R^, TET^R^, CRO^R^, CFM^R^, and CAZ^R^
S.E 58	AMP^R^, AMX^R^, CHL^R^, CIP^R^, NAL^R^, TET^R^, CAZ^R^, GEN ^R^, and SXT ^R^
S.E 91	AMP^R^, AMX^R^, CHL^R^, CIP^R^, NAL^R^, and GEN ^R^

Note: S.E: *Salmonella enteritidis*; R: resistant; AMP: ampicillin; AMX: amoxicillin; CHL: chloramphenicol; CIP: ciprofloxacin; NAL: nalidixic acid; TET: tetracycline; CRO: ceftriaxone; CFM: cefixime; CAZ: ceftazidime; GEN: gentamicin; SXT: sulfamethoxazole.

An agar diffusion assay was performed following the previous method with slight modifications [1,15,16,25]. In brief, ∼0.5 McFarland inoculum of *Salmonella enteritidis* was swabbed on Mueller–Hinton (MH) agar. Wells were created, sealed with molten agar, and filled with cell-free supernatants (CFSs; 80–100 µL) of lactobacilli. CFSs were also tested after neutralization using NaOH (1 mol L^−1^) and inactivation of H_2_O_2_ using catalase. MRS broth was used as a negative control in the test. After 24 h incubation at 37°C, the diameter of inhibition zones was measured in millimeters.

### 2.3 *In vitro* probiotic properties of lactobacilli

All the tests essential for the assessment of probiotic potential were performed in accordance with the previous related studies [10,21,26].

#### 2.3.1 Tolerance to acidic pH and ox-bile

Acid tolerance of lactobacilli was determined as described by Asghar et al. [26], with some modifications. Briefly, lactobacilli (3 × 10^7^ CFU) were exposed to normal saline having pH 2, 3, 4, and 7, adjusted with HCl (0.1 mol L^−1^)/NaOH (1 mol L^−1^). After 3 h incubation at 37°C, 20 µL of pH-exposed lactobacilli (6 × 10^6^ CFU) were re-cultured in 200 µL of MRS broth (pH = 7) supplemented with L-cysteine (0.5 g L^−1^), using a 96-well microtiter plate. The plate was then incubated at 37°C. The pH tolerance was estimated by calculating the difference in optical density (OD) values at 24 h and 0 min, recorded at 630 nm using a Rayto Microtiter plate reader (RT-2100C). To monitor the ability of lactobacilli to survive acidic conditions, their growth was observed in MRS broth with pH adjusted to 3, 4, and 7. The bacterial growth was indicated by an increase in OD values after 24 h incubation.

To check tolerance to ox-bile, lactobacilli (3 × 10^7^ CFU) were inoculated in 200 µL of MRS broth supplemented with 0.3, 1.0, and 1.8% purified ox-bile (Hi-Media) and l-cysteine (0.5 g L^−1^) [27,28]. After 24 h incubation at 37°C, tolerance was assessed by calculating the difference in OD values at 0 min and 24 h.

#### 2.3.2 Activity against other gut pathogens

The activity of isolates against *Campylobacter jejuni* (ATCC33291^TM^), *E. coli* ZQN9, and *Salmonella* Gallinarum G1 was determined by the agar well diffusion method as described earlier.

#### 2.3.3 Antibiotic susceptibility pattern

Minimum inhibitory concentrations (MICs) of ampicillin, amoxicillin, chloramphenicol, vancomycin, erythromycin, tetracycline, penicillin, ciprofloxacin, lincomycin, streptomycin, doxycycline, and levofloxacin against lactobacilli (*n* = 24) were determined by the broth microdilution method in 96-well microtiter plates as demonstrated by Saleem [29]. Two-fold dilutions of antibiotics (0.25–128 μg mL^−1^) were prepared in the LAB susceptibility test medium using microtiter plate wells. Inoculums of lactobacilli (∼1 McFarland) were prepared by suspending their fresh growth in normal saline, followed by dilution (1/10^3^) in MRS broth. Then, the doubly diluted antibiotics (50 μL) were inoculated with prepared inoculum (100 μL, ∼3 × 10^4^ CFU). Microtiter plates were then incubated at 37°C for 24 h in anaerobic conditions, followed by observation of visible growth or turbidity. Minimum concentrations of antibiotics inhibiting the visible growth of isolates were considered as MICs. Isolates were designated as resistant, intermediate, and sensitive to the tested antibiotics following the breakpoints given by the European Food Safety Authority (EFSA) [30], except the breakpoints for ciprofloxacin and levofloxacin were adopted from the Clinical and Laboratory Standards Institute.

#### 2.3.4 Auto-aggregation and Co-aggregation assays

Auto-aggregation and co-aggregation abilities of lactobacilli were determined according to the procedure of Angelov [31]. Briefly, ∼1 McFarland suspension of freshly grown *Lactobacillus* and *Salmonella enteritidis* were incubated alone and in combination (1:1) at 37°C. Afterward, OD of 0.2 mL supernatant was recorded at 0 min, 1, 2, and 16 h. Percentage auto-aggregation and co-aggregation were calculated using the same formulae that were used by Angelov [31].

### 2.4 *Salmonella enteritidis* inhibition in broth cultures

A broth inhibition test was performed by adapting the procedure used by Khan et al. [21]. Suspensions (100 µL) of *Salmonella enteritidis* (0.5 McFarland) and *Lactobacillus* (1 McFarland) were mixed in skim milk broth (10 mL) and incubated at 37°C. *Salmonella enteritidis* and lactobacilli were enumerated from this mixture at different intervals, using *Salmonella Shigella* and MRS agar, respectively.

### 2.5 *In vivo* evaluation of probiotic lactobacilli for prevention of *Salmonella enteritidis* in mice

The probiotic potential of selected lactobacilli against *Salmonella enteritidis* was evaluated in mice using the previous protocol with some modifications in experimental design [32]. *In vivo* experiments were conducted in compliance with the institutional ethical guidelines. Ethical approval was obtained from the institutional ethical review committee of UVAS, Lahore (approval No. 408; dated: 22/04/2019). Healthy conventional Balb/c mice were purchased from the University of Health Sciences, Lahore, and moved to the cages placed in controlled mouse rooms of the Institute of Microbiology, UVAS, at 22 ± 2°C temperature, with 65% humidity and alternate 12 h periods of light and dark. All the males mice at 8 weeks of age were divided into 5 groups, each with 5 mice. They were supplied with potable water and commercial standard fat diet *ad libitum* and housed in cages (filled with wood shavings). Negative control mice were administered with sterilized normal saline. The positive control group received only *Salmonella enteritidis*. The experimental groups were orally administered with a probiotic dose (0.6 × 10^8^ CFU), using orogastric gavage, for 17 days. Experimental and positive control groups were additionally challenged (twice) with *Salmonella enteritidis* (D) ATCC13076^TM^ (∼10^7^ CFU mL^−1^) on the 7th and 15th day of the experiment. Fresh fecal samples were collected using sterile forceps and diluted (ten-fold) using sterile normal saline. The diluted feces (0.2 mL) were spread on both the *Salmonella Shigella* agar and MRS agar supplemented with vancomycin (100 µg mL^−1^) and fluconazole (100 µg mL^−1^), separately. The plates were then incubated at 37°C for 24–48 h. *Lactobacillus* counts and *Salmonella* counts were then performed.


**Ethical approval:** The research related to animal use has been complied with all the relevant national regulations and institutional policies for the care and use of animals.

### 2.6 Statistical analysis

All analyses were carried out using one-way ANOVA followed by Tukey’s test, using GraphPad Prism 5.0 software. A *p-*value < 0.05 was considered significant.

## 3 Results

### 3.1 Isolates identified as *Lactobacillus* spp.

*Lactobacillus* isolates (*n* = 24) were purified from 30 samples, 9 from yogurt and 15 from feces of healthy human infants who were fed on breast milk and did not receive any previous treatment with probiotics and antibiotics. All isolates were Gram-positive rods and catalase-negative. All 24 isolates were confirmed as lactobacilli using *Lactobacillus* genus-specific polymerase chain reaction. Isolates were confirmed as *L. fermentum* (FY1 [MN153531], FY3 [MN153532], FY4 [MN153533], IL2 [MN153536 – 16S rDNA; MN161530 – 16S–23S inter-spacer], and IL5 [MN161531]), *L. delbrueckii* (FY6 [MN153534] and FY7 [MN153535]), *Lactobacillus* sp. (IL7), *L. gasseri* (IL12 [MN153537 – 16S rDNA; MN161532 – 16S–23S inter-spacer]), and other species of *Lactobacillus*.

### 3.2 Anti-microbial effect of lactobacilli against *S. enteritidis*

**Figure 1:**
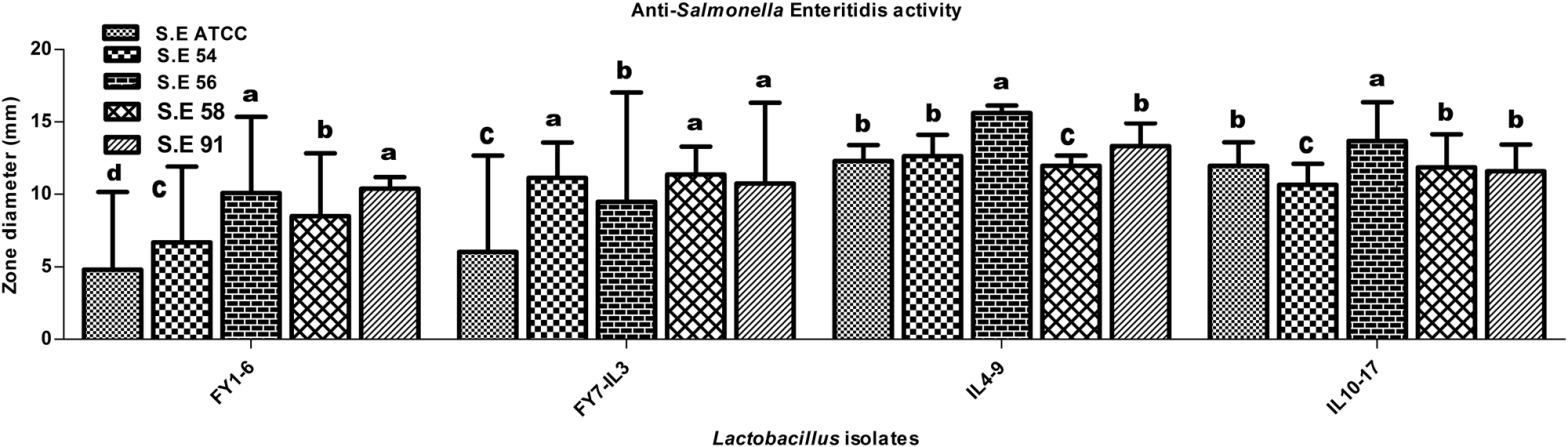
Anti-*Salmonella enteritidis* activity (mean value ± SD) of CFSs of lactobacilli. No zones of inhibition were detected in neutralized CFSs except a few with ≤8.6 ± 0.5 mm zone diameter. Inactivation of H_2_O_2_ did not affect the activity of supernatants. S.E: *Salmonella enteritidis*; SD: Standard Deviation. a, b, c, and d indicate statistically significant difference (*p* < 0.05) among different bars of the same group.

The activity of lactobacilli against *Salmonella enteritidis* was indicated by ≤16.6 ± 0.5 mm inhibition zones against salmonellae, determined by a well diffusion assay (Figure 1).

### 3.3 *In vitro* probiotic properties of lactobacilli

**Figure 2:**
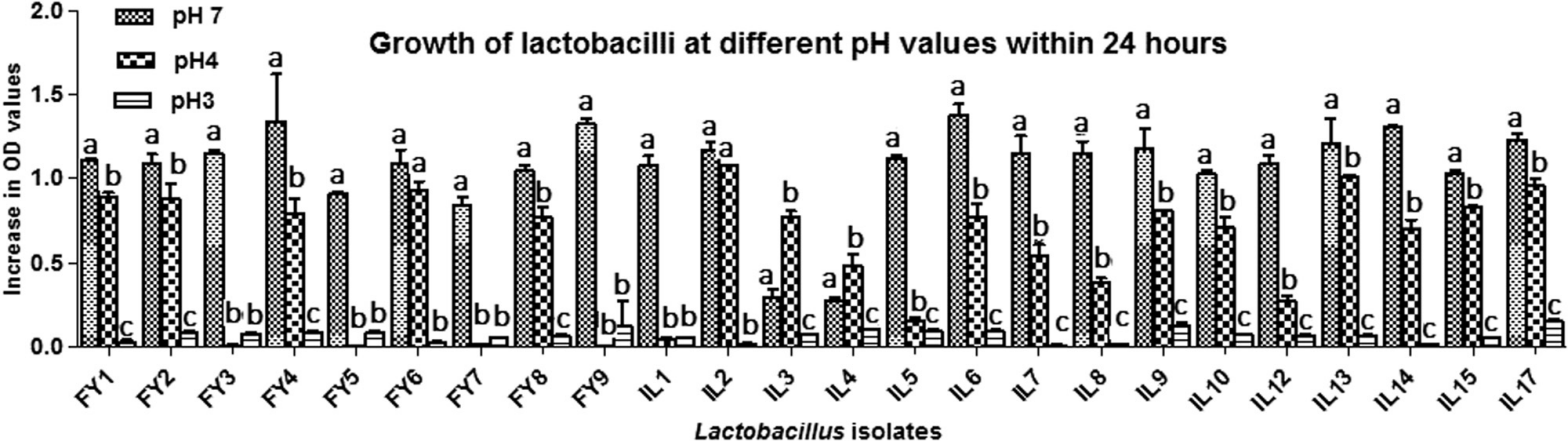
Growth of lactobacilli at different pH values within 24 h. SD: standard deviation; OD: optical density. a, b, and c indicate statistically significant difference (*p* < 0.05) among different bars of the same group.

None of the isolates showed growth at pH 3, except FY9, IL4, IL9, and IL17 which showed an increase in OD (≤0.148 ± 0.015). Most lactobacilli showed better growth at pH 4 (increase in OD ≤ 1.076 ± 0.006) compared to pH 3, except FY3, FY5, FY7, FY9, and IL1. The IL2 and IL13 showed the highest growth at pH 4 (Figure 2).

The isolates also showed varying degrees of tolerance to pH 4 (increase in OD ≤ 1.291 ± 0.068) for 3 h, except FY1, FY5, and IL6. Only 9 isolates tolerated pH 3 (increase in OD ≤ 1.359 ± 0.4) for 3 h, as shown in Table 2. Isolates FY3 and IL15 additionally tolerated pH 2 (increase in OD ≤ 1.067 ± 0.05) for 3 h. In short, lactobacilli were more tolerant to pH 4 compared to lower pH values.

**Table 2:** Tolerance of lactobacilli to different pH values for 3 h

Sr No	Isolate	Tolerance for 3 h (increase in OD)			
		pH 7	pH 4	pH 3	pH 2
1	FY1	0.716 ± 0.5^a^	0.015 ± 0.01^b^	1.017 ± 0.005^a^	0.034 ± 0.002^b^
2	FY2	1.063 ± 0.04^a^	1.061 ± 0.08^a^	0.982 ± 0.01^a^	0.076 ± 0.003^b^
3	FY3	0.916 ± 0.04^a^	1.028 ± 0.05^a^	0.07 ± 0.008^b^	0.609 ± 0.5^b^
4	FY4	1.008 ± 0.07^a^	0.942 ± 0.06^a^	0.068 ± 0.008^b^	0.062 ± 0.008^b^
5	FY5	1.140 ± 0.09^a^	0.006 ± 0.01^b^	0.624 ± 0.02^b^	0.202 ± 0.1^c^
6	FY6	1.190 ± 0.16^a^	0.466 ± 0.65^b^	0.058 ± 0.003^b^	0.046 ± 0.01^b^
7	FY7	1.234 ± 0.13^a^	0.043 ± 0.021^b^	0.059 ± 0.005^b^	0.238 ± 0.313^b^
8	FY8	0.72 ± 0.52^a^	0.65 ± 0.52^a^	0.051 ± 0.003^a^	0.049 ± 0.006^a^
9	FY9	0.909 ± 0.38^a^	1.29 ± 0.12^a^	0.047 ± 0.005^b^	0.046 ± 0.001^b^
10	IL1	0.047 ± 0.06^a^	1.073 ± 0.02^b^	1.359 ± 0.4^b^	0.021 ± 0.002^a^
11	IL2	0.812 ± 0.66^a^	1.024 ± 0.07^a^	0.98 ± 0.03^a^	0.027 ± 0.003^b^
12	IL3	1.136 ± 0.04^a^	1.07 ± 0.05^a^	0.075 ± 0.05^b^	0.029 ± 0.001^b^
13	IL4	1.045 ± 0.02^a^	1.078 ± 0.11^a^	0.025 ± 0.02^b^	0.02 ± 0.01^b^
14	IL5	1.120 ± 0.07^a^	1.065 ± 0.06^a^	1.045 ± 0.04^a^	0.201 ± 0.1^b^
15	IL6	1.124 ± 0.07^a^	1.159 ± 0.22^a^	0.053 ± 0.01^b^	0.013 ± 0.013^b^
16	IL7	1.215 ± 0.21^a^	0.069 ± 0.006^b^	0.543 ± 0.03^c^	0.043 ± 0.04^b^
17	IL8	1.077 ± 0.21^a^	1.148 ± 0.12^a^	1.096 ± 0.07^a^	0.024 ± 0.001^b^
18	IL9	1.147 ± 0.01^a^	1.291 ± 0.068^b^	0.992 ± 0.01^c^	0.023 ± 0.03^d^
19	IL10	0.405 ± 0.54^a^	1.136 ± 0.09^b^	0.090 ± 0.008^a^	0.016 ± 0.01^a^
20	IL12	1.021 ± 0.02^a^	1.19 ± 0.18^a^	1.057 ± 0.003^a^	0.013 ± 0.02^b^
21	IL13	1.166 ± 0.01^a^	0.482 ± 0.7^b^	0.078 ± 0.002^b^	0.012 ± 0.01^b^
22	IL14	1.115 ± 0.06^a^	1.083 ± 0.09^a^	0.304 ± 0.385^b^	0.004 ± 0.03^b^
23	IL15	1.177 ± 0.18^a^	1.277 ± 0.24^a^	1.121 ± 0.01^a^	1.067 ± 0.05^a^
24	IL17	0.893 ± 0.1^a^	0.934 ± 0.04^a^	0.059 ± 0.007^b^	0.02 ± 0.01^b^

Note: a, b, c, and d indicate statistically significant difference (*p* < 0.05) among different columns of the same row. SD: standard deviation; OD: optical density. Increase in OD = OD after 24 h – OD at 0 min.

**Figure 3:**
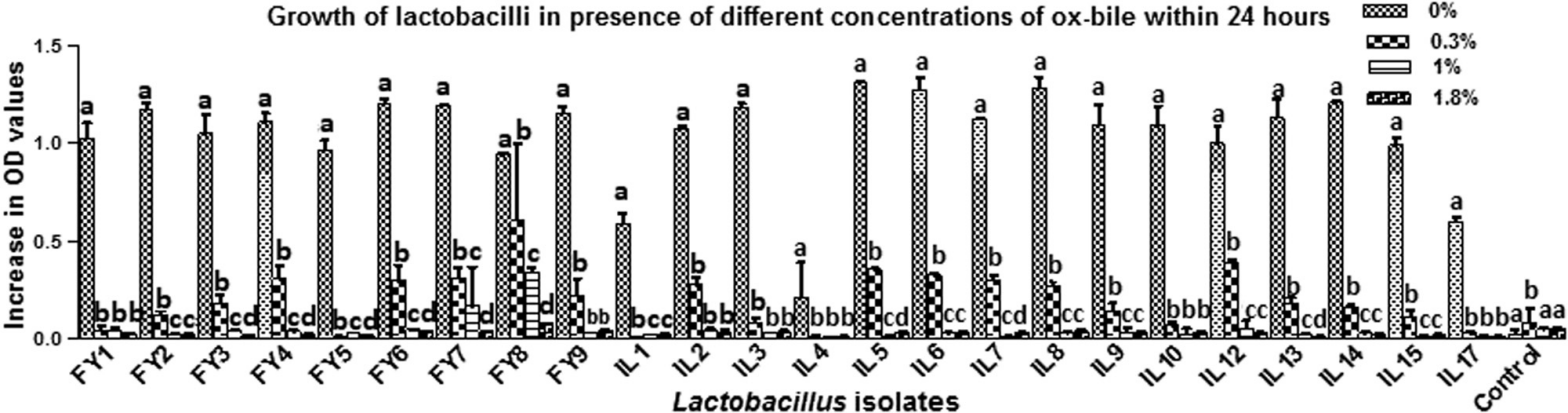
Growth of lactobacilli in MRS broth supplemented with different concentrations of ox-bile within 24 h. a, b, c, and d indicate statistically significant difference (*p* < 0.05) among different bars of same group; SD: dtandard deviation.

*Lactobacillus* isolates (*n* = 18) showed good growth rates in the presence of 0.3% ox-bile (increase in OD ≤ 0.603 ± 0.4), and 1% ox-bile (increase in OD ≤ 0.339 ± 0.02) as indicated in Figure 3.

**Figure 4:**
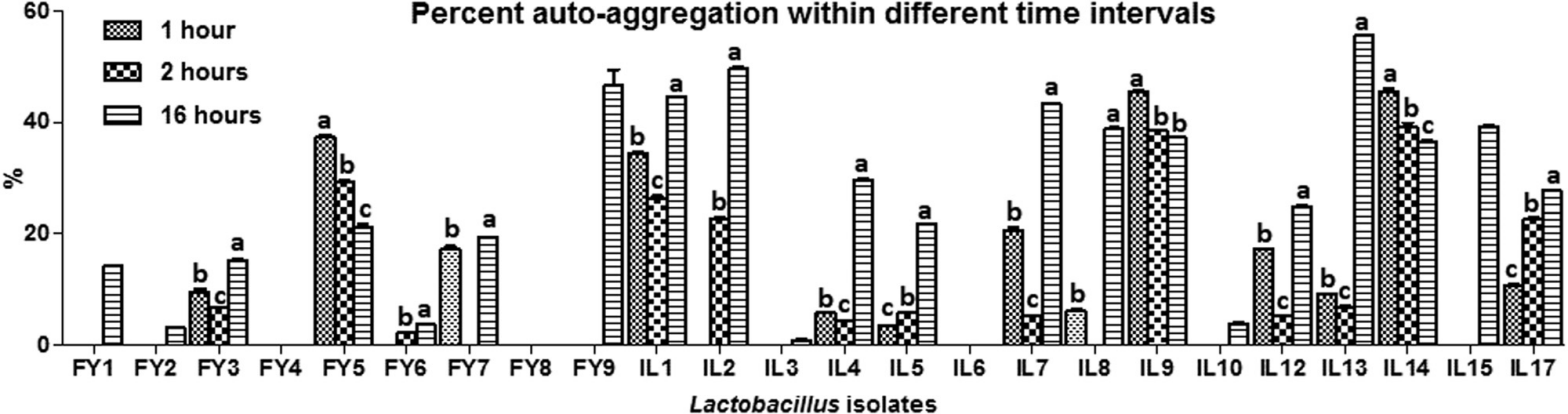
Auto-aggregation of lactobacilli within different time intervals.

**Figure 5:**
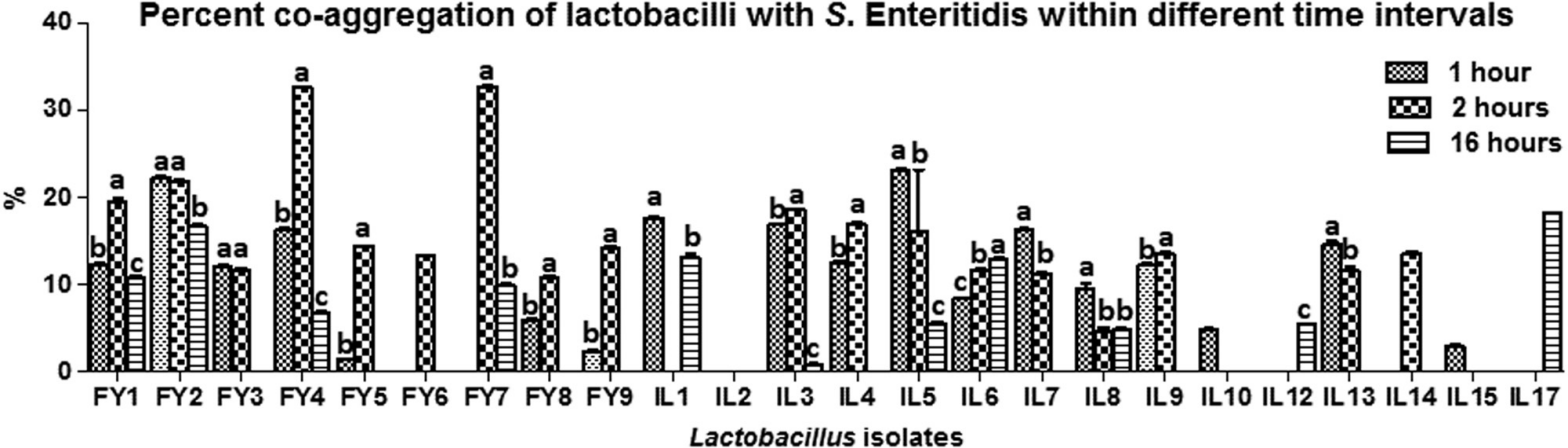
Co-aggregation of lactobacilli with *Salmonella enteritidis* within different time intervals.

All the isolates had variable auto-aggregation and co-aggregation (with *Salmonella enteritidis*) capabilities, ranging between 0.8 ± 0.2 and 55.6 ± 0.1% and 0.8 ± 0.1 and 32.7 ± 0.2%, respectively (Figures 4 and 5).

The safety profile of lactobacilli indicated sensitivity to all the tested antibiotics except vancomycin, ciprofloxacin, lincomycin, and streptomycin (Table 3). Lactobacilli also had variable activity against other gut pathogens, including *Campylobacter jejuni* ATCC (≤15.3 ± 0.5 mm), *E. coli* ZQN9 (≤11.3 ± 0.5 mm), and *Salmonella* Gallinarum G1 (≤12 ± 0 mm).

**Table 3:** Antibiotic resistance pattern of lactobacilli

Sr No.	Isolates	Resistance profile
1	FY1	VAN^R^, CIP^R^, and STR^R^
2	FY2	VAN^R^, CIP^R^, and STR^R^
3	FY3	VAN^R^, CIP^R^, and STR^R^
4	FY4	VAN^R^, CIP^R^, and STR^R^
5	FY5	VAN^R^, CIP^R^, and STR^R^
6	FY6	CIP^R^
7	FY7	CIP^R^ and STR^R^
8	FY8	VAN^R^, CIP^R^, and STR^R^
9	FY9	VAN^R^, CIP^R^, and STR^R^
10	IL1	VAN^R^, CIP^R^, LCM^R^, and STR^R^
11	IL2	VAN^R^, CIP^R^, and LCM^R^
12	IL3	VAN^R^, CIP^R^, LCM^R^, and STR^R^
13	IL4	VAN^R^, CIP^R^, LCM^R^, and STR^R^
14	IL5	VAN^R^, LCM^R^, and STR^R^
15	IL6	VAN^R^, CIP^R^, LCM^R^, and STR^R^
16	IL7	VAN^R^, CIP^R^, LCM^R^, and STR^R^
17	IL8	VAN^R^, CIP^R^, LCM^R^, and STR^R^
18	IL9	VAN^R^, CIP^R^, LCM^R^, and STR^R^
19	IL10	CIP^R^, LCM^R^, and STR^R^
20	IL12	VAN^R^, CIP^R^, and LCM^R^
21	IL13	VAN^R^, CIP^R^, and STR^R^
22	IL14	VAN^R^, CIP^R^, and STR^R^
23	IL15	VAN^R^, CIP^R^, and STR^R^
24	IL17	VAN^R^, CIP^R^, and STR^R^

Note: R: resistant; VAN: vancomycin; CIP: ciprofloxacin; STR: streptomycin; LCM: lincomycin.

### 3.4 *In vitro* and *in vivo* activities of probiotic lactobacilli against *S. enteritidis*

All the selected isolates (*L. fermentum* IL5, *L. fermentum* IL2, and *L. gasseri* IL12) resulted in a 100% reduction in *S. enteritidis* in broth (Table 4), followed by efficient colonization in mouse guts, as indicated by increased *Lactobacillus* counts (8.77 ± 0.02 log_10_ CFU mL^−1^) in mice feces (Table 5). No *Salmonella* was detected in mice feces of IL5 and IL12 groups at the end of the trial. On the other hand, there was a 2 log reduction in *Salmonella* counts in the case of IL2 group. There was no significant reduction in *Salmonella* counts in mice feces of positive and negative control groups. Within a week, there was 2.02, 3.37, and 4.32 mean log_10_ reduction in *Salmonella* counts in mice feces by IL2, IL5, and IL12, respectively. In other words, the tested isolates (IL2, IL5, and IL12) showed 99.06, >99.9, and >99.99% reduction in *Salmonella* counts, respectively. Fortunately, no mortality was observed during the *in vivo* experiment.

**Table 4:** Reduction in *Salmonella enteritidis* in co-culture with selected probiotics

Lactobacilli	Log_10_ CFU mL^−1^ (mean value ± SD) of *Salmonella enteritidis*				*Salmonella enteritidis* reduction at 48 h (%)
	0 min	4 h	24 h	48 h	
IL2	6.35 ± 0.03^a^	8.92 ± 0.02^c^	7.65 ± 0.03^b^	ND	100
IL5	5.92 ± 0.02^a^	7.23 ± 0.02^c^	6.55 ± 0.05^b^	ND	100
IL12	4.91 ± 0.03^a^	8.82 ± 0.02^c^	6.68 ± 0.02^b^	ND	100

Note: a, b, and c indicate statistically significant difference (*p* < 0.05) among different columns of the same row; ND: not detected; SD: standard deviation; CFU: colony-forming unit.

**Table 5:** Effect of probiotic lactobacilli on *Salmonella enteritidis* colonization in mice

Group	log_10_ CFU mL^−1^ (mean value ± SD)								
	Day 1		Day 8		Day 10		Day 16		Day 17
	S.E	*Lactobacillus*	S.E	*Lactobacillus*	S.E	*Lactobacillus*	S.E	*Lactobacillus*	S.E
NC	—	—	2.68 ± 0.02^a^	7.33 ± 0.02^a^	2.57 ± 0.02^a^	8.16 ± 0.03^a^	2.68 ± 0.02^a^	7.57 ± 0.02^a^	2.31 ± 0.03^a^
PC	—	—	3.35 ± 0.05^b^	8.21 ± 0.01^b^	3.47 ± 0.02^b^	7.57 ± 0.02^b^	4.82 ± 0.02^b^	3.6 ± 0.07^b^	4.32 ± 0.02^b^
IL2	ND	3.16 ± 0.01^a^	4.94 ± 0.02^c^	7.78 ± 0.02^c^	2.74 ± 0.02^c^	7.91 ± 0.01^c^	2.95 ± 0.02^c^	7.56 ± 0.02^a^	2.30 ± 0.02^a^
IL5	ND	3.44 ± 0.04^b^	ND	8.77 ± 0.02^d^	ND	8.12 ± 0.02^a^	ND	8.05 ± 0.05^c^	ND
IL12	2.83 ± 0.02	4.31 ± 0.07^c^	2.62 ± 0.02^a^	7.82 ± 0.02^c^	5.61 ± 0.02^d^	8.67 ± 0.02^d^	2.48 ± 0.01^d^	8.54 ± 0.04^d^	ND

Note: a, b, c, and d indicate statistically significant difference (*p* < 0.05) among different rows of the same column; CFU: colony-forming units; SD: standard deviation; S.E: *Salmonella enteritidis*; NC: negative control; PC: positive control.

## 4 Discussion

The frequent use of antibiotics against *Salmonella enteritidis* has resulted in antibiotic-resistant salmonellae [9,23]. One of the recently proposed sustainable alternatives to antibiotics for the prevention of *Salmonella enteritidis* is the use of *Lactobacillus* as probiotics [33]. Various dietary probiotic microbes, including *L. rhamnosus*, *L. reuteri, L. casei, L. acidophilus, E. coli*, *Bifidobacterium*, *Enterococci faecium,* and *Saccharomyces boulardii* have been successfully investigated for medicinal use, either as single strain or as mixed cultures [34]. *Lactobacillus* is a natural resident of human, animal, and avian gastrointestinal tracts (GIT) and has been isolated from all parts of intestines, plants, and fermented foods [21,23,26,35]. Other studies isolated probiotic *L. fermentum, L. reutri, L. gallinarum, L. plantarum,* and *L. brevis* from homemade yogurt, Brazilian food products, and indigenous poultry [36,37]. Likewise, the current research isolated the local lactobacilli not only from yogurt but also from breastfed babies. As these lactobacilli are of human origin and were already a part of microflora, they may exert better probiotic effects on human GIT. Likewise, lactobacilli have also been isolated from the stool of breastfed healthy babies in China using the same strategy [24]. *Ligilactobacillus salivarius* CECT 5713, isolated from a breastfed 1-month-old infant, proved to be a potential probiotic strain [38]. In a recent study, Dobreva et al. [39] isolated *Lactiplantibacillus plantarum* strains, having activity against *S*. Typhimurium, from breast milk and tested their efficiency in simulated real conditions in the food chain. *L. gasseri* has also been reported as an important component of vaginal microflora [40]; thus, *L. gasseri* IL12 isolated from the stool of breastfed infants in the present study may be a representative of the mother’s reproductive tract flora.

Many studies have investigated the effects of probiotics against *Salmonella* [21,26,41,42]. Usually, *in vitro* investigations are carried out before *in vivo* studies for probiotic screening. However, the results of all the *in vitro* assays are not always consistent with the outcomes of *in vivo* experiments. In order to screen anti-*Salmonella enteritidis* lactobacilli, a well diffusion assay was used in the present study, while Kowalska et al. [43] used the agar slab method for the same purpose. Moreover, Zhou et al. [44] showed the anti-*Salmonella* mode of action of natural L-phenyl lactic acid purified from *L. plantarum*. Another study showed the successful inhibition of *Salmonella* invasion to intestinal epithelium HT29 cells by probiotic lactobacilli (*L. salivarius* and *L. agilis*) of porcine origin [45]. Recently, anti-fungal and anti-aflatoxigenic lactobacilli have also been screened [46].

To reach the intestine, microorganisms must survive acidity stress (pH 1.5–2) in the stomach. *Lactobacillus* is capable of using a proton antiport system for pH maintenance in a wide range of environments [47]. The ability of an organism to grow in MRS broth at acidic pH or to tolerate acid stress is often used for probiotic selection [24]. Current research showed visible growth of *Lactobacillus* isolates at both pH 4 and pH 7. In addition, tolerance to acid was also monitored by checking survival after exposure to acid stress without any nutrition. For the said purpose, normal saline (0.89% NaCl) was preferentially used because it provides osmotic protection to bacterial cells. Normal saline normally has a pH of around 5.5. To expose isolates to acid stress without any nutrition, the pH of saline was adjusted to 3, 4, and 7 using HCl (0.1 mol L^−1^)/NaOH (1 mol L^−1^). The similar tolerant behavior of lactobacilli has also been previously identified by Khan et al. [21] and Asghar et al. [26], employing the same strategy. Mostly, researchers used to record log counts of survivors after exposure to acid or bile. In contrast, the present study measured the OD, with increased OD values confirming the capability of isolates to survive in/after stressed conditions. According to the previous report, lactobacilli showed 41% survival after 90 min of exposure to pH 2 and 3 [26]. Similarly, Aazami et al. [41] reported high growth rates of *Limosilactobacillus reuteri* and *Ligilactobacillus salivarius* at pH 2.5. G-Alegría et al. [48] also showed that *Lactiplantibacillus plantarum* isolates could grow well at pH 3.2. To establish in the gut, lactobacilli should possess the ability to grow in the presence of at least 0.3% bile salts [26,49]. Lactobacilli isolated in present study showed growth in the presence of 0.3% ox-bile (increase in OD ≤ 0.603 ± 0.4), and 1% ox-bile (increase in OD ≤ 0.339 ± 0.02). Previously reported probiotics also showed a similar degree of resistance [26]. The greater resistance of isolates against 0.3% ox-bile compared to 1% and 1.8% agrees with previous findings [21]. A high degree of bile tolerance in *L. fermentum, L. plantarum,* and *L. brevis* has also been examined in a previous study [37].

The auto-aggregation capability of a probiotic strain is necessary for attachment to the gut epithelium. Whereas co-aggregation provides a barrier effect against pathogen colonization. The present study indicated variable auto-aggregation and co-aggregation (with *Salmonella enteritidis*) capabilities of lactobacilli, ranging between 0.8 ± 0.2 and 55.6 ± 0.1% and 0.8 ± 0.1 and 32.7 ± 0.2%, respectively. Several prior research works also reported similar effects of lactobacilli against *Salmonella* [21,26,41].

The EFSA has declared lactobacilli as generally recognized as safe microorganisms [49]. However, antibiotic susceptibility of food origin probiotics should be determined to prevent the transfer of potential anti-microbial resistance traits to commensal flora of animals, humans, and pathogens [50]. *Lactobacillus* sp. is generally sensitive to protein synthesis and cell wall synthesis inhibitors, except aminoglycoside and vancomycin, respectively. Whereas lactobacilli are mostly resistant to DNA synthesis inhibitors [19]. All lactobacilli isolated in the present study were sensitive to most of the tested antibiotics. This study also revealed vancomycin, ciprofloxacin, and streptomycin-resistant lactobacilli (Table 2) as reported previously [19]. Criteria for selecting probiotic strain also involve the activity against gut pathogens. Anti-*Salmonella* lactobacilli also showed activity against *Campylobacter jejuni* ATCC, *E. coli* ZQN9, and *Salmonella* Gallinarum G1. Recent reports are also available, indicating 6.5–12 mm inhibition zone radii [51,52,53]. In this way, probiotic bacteria suppress the growth of undesirable bacteria in the gut, resulting in the stabilization of the digestive system [54].

Surface layer proteins of *Lactobacillus* and adhesins (lectins) found in the bacterial cell wall and intestinal epithelium favor the colonization of *Lactobacillus* in the gut and the competitive exclusion of pathogen [55]. Due to this character, oral administration of probiotic potential lactobacilli can reduce *Salmonella enteritidis* counts in the intestine, resulting in reduced infections. The results of co-culture experiments indicated the ability of lactobacilli to compete for limiting nutrients, while the growth of *Salmonella enteritidis* was completely inhibited after 48 h. A previous study also obtained similar results from a co-culture experiment just after 10 h [56]. Overall, ≥99.06 percent reduction and ≤4.32 Mean log_10_ reduction in *Salmonella* counts from mice feces indicated the inability of *Salmonella* to adhere to the gut in the presence of *Lactobacillus* isolates. This may result from lactic acid production in the gut or competitive exclusion of *Salmonella enteritidis*. Another previous research showed the anti-microbial effect of *Lactobacillus* against *Salmonella* in a mouse model [42]. Recently, yeasts have also been subjected to co-aggregation with *Salmonella*, antagonism to salmonellosis, and *in vivo* survival, which presented high co-aggregation and satisfactorily survived the passage through the GIT of mice [57]. These *Lactobacillus* isolates can be proposed as potential probiotics to prevent *Salmonella enteritidis* infections in humans but only after confirmation using further experiments. For example, a recent study indicated the probiotic effect of *Artemisia argyi* broth fermented with *Lactiplantibacillus plantarum* against *Salmonella Typhimurium* after showing *in vitro* growth inhibition, reduction in adherence, and invasion of HT-29 cell line, decreased bacterial load not only in GIT but also in internal organs (spleen, liver, and mesenteric lymph nodes) of mice, decreased level of pro-inflammatory cytokines in serum, and improved epithelial barrier integrity [58]. In the current work, lactobacilli also showed significant *in vitro* activity against salmonellae of poultry origin; thus, these can also be evaluated in poultry birds like a previous study which indicated probiotic *Bacillus licheniformis* and *Bacillus subtilis* as tools for reducing *Salmonella* load in broiler GIT [59]. A significant reduction in mean *Salmonella* counts in feces has also previously been observed in probiotic-treated animals [60]. Various *in vitro* models suggested the blockage of *Salmonella* binding sites by lactobacilli. For example, *L. fermentum* was found to cause an effective reduction in the colonization of *Salmonella* Pullorum by 77%, whereas *Ligilactobacillus animalis* inhibited the adhesion of *Salmonella* Pullorum*, Salmonella enteritidis*, and *Salmonella* Gallinarum to host epithelial fragments by 90, 88, and 78%, respectively [61]. An antagonistic effect of *Lactobacillus acidophilus* LA10 against *Salmonella enteritidis* SE86 was also demonstrated in conventional mice upon the oral administration of 10^8^ CFU of each of SE86 and LA10 [32]. Recently, Liu et al. [42] also found the reduction in lethal effects of *Salmonella* by pretreatment of mixed *Lactobacillus* strains in a mouse model, especially *L. plantarum* exhibited remarkable performance with better preventive effects. During *in vivo* evaluations, the effect of lactobacilli on other parameters like body weight gain, feed conversion ratio, phytate solubilization, enhanced immune response, and the effects on gut morphology and intestinal absorption capacity have also been examined previously [14,26,36,52]. Further research may include studying the factors affecting the activity of lactobacilli, such as the latest research showing that xylooligosacharides promote the anti-*Salmonella* activity of *L. plantarum* [62].

The present study is the first step of a multistep project to develop indigenous probiotics against *Salmonella enteritidis* to prevent and mitigate *Salmonella enteritidis*-associated foodborne infections. A lot of probiotic products are imported in Pakistan but none of these use the beneficial strains of the local environment. It is imperative to explore the local strains for added benefits. Thus, current work provided characterized *L. fermentum* IL2, *L. fermentum* IL5, and *L. gasseri* IL12, resulting in the availability of local probiotic lactobacilli in Pakistan, which can be considered potential ingredients for the mass production and further development of probiotic products for humans and poultry, after further evaluations. Thus, it will help to cope with the insufficiency of local probiotic products in Pakistan, and consumers’ health will be improved. In this way, by using probiotics as an alternative, the emergence of antibiotic-resistant strains could also be controlled. In the next phase, we will explore other benefits of these strains, i.e., immunomodulatory effects and suitable foods as the delivery vehicle.
